# Supplementary material for: Synthesis, Stability, and Bioavailability of Nicotinamide Riboside Trioleate Chloride
Source: Nutrients. 2021 Dec 27;14(1):113. doi: 10.3390/nu14010113 (PMC8746687; doi:10.3390/nu14010113)
Supplement: Supplementary file 1 [file nutrients-14-00113-s001.zip › nutrients-1494833-supplementary.pdf]

Synthesis, stability, and bioavailability of nicotinamide riboside trioleate chloride

Amin Zarei[a]†, Leila Khazdooz[a]†, Sara Madarshahian[a], Mojtaba Enayati[a], Imann Mosleh [a], Tiantian Lin [a], Bing Yan[b], Gerhard Ufheil[b], Timothy James Wooster[c], Alireza Abbaspourrad[a]*

[a] Department of Food Science, College of Agriculture and Life Sciences, Cornell University, Ithaca 14853, NY, USA

[b] Nestlé Product Technology Center, Nestlé Health Science, Bridgewater, NJ 08807, USA

[c] Nestlé Research, Lausanne 26, Switzerland

†These authors contributed equally to this work.

Corresponding Author; Email: Alireza@cornell.edu (A.A.)

Characterization of NRTOCl

Fourier Transform Infrared (FTIR)

The FTIR of NRTOCl has two bands at 3289 and 3123 cm^{-1} are asymmetric and symmetric stretching bonds of NH_2 in the amide functional group. Alkene and aromatic C-H stretching vibrations appear around 3005 cm^{-1}. The exitance of two bands at 2922 and 2853 cm^{-1} is attributed to asymmetric and symmetric stretching vibrations of aliphatic C-H. A strong band at 1744 cm^{-1} demonstrates the carbonyl of ester groups. The carbonyl of the amide functional group appears at 1689 cm^{-1}. The band at 1622 cm^{-1} is evidence of the presence of the C=C bond. Two bands at 1458 and 1379 cm^{-1} show the bending vibrations of the methylene and methyl groups respectively. A broad band between 1100-1250 cm^{-1} is attributed to the C-O stretch of the ester groups. The bands at 677, 721 and 915 cm^{-1} show the alkene and aromatic C-H bending vibrations (Figure S-1).

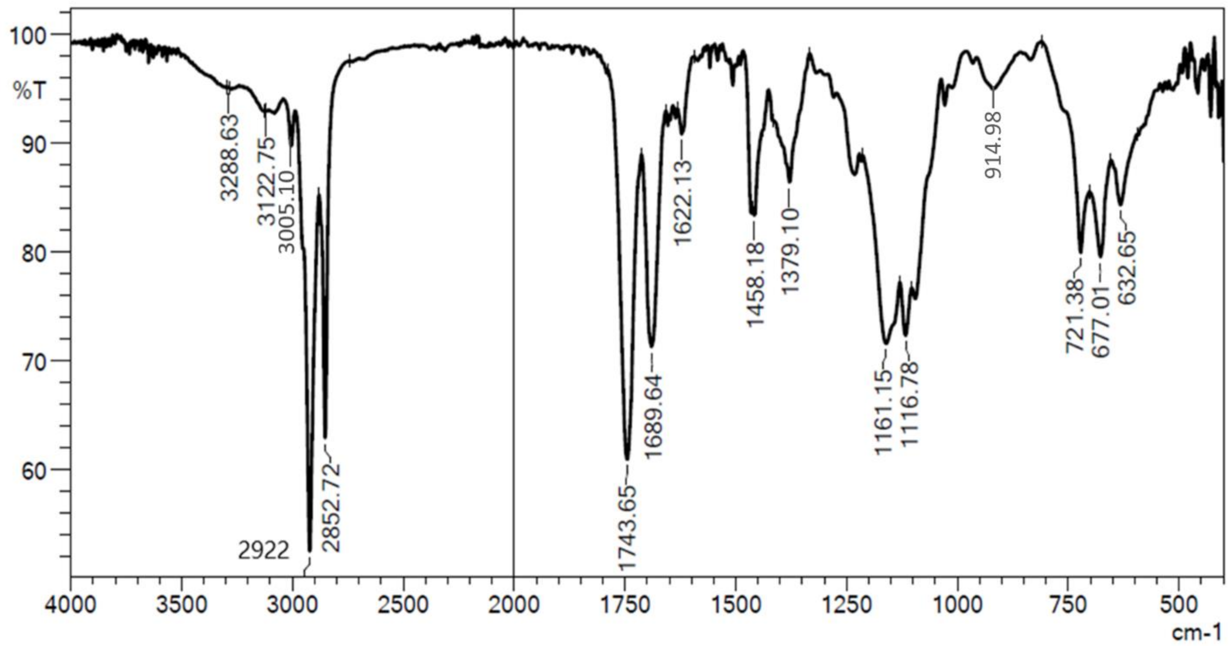

Figure S-1. ATR FT-IR of NRTOCl.

500 MHz ^{1}H NMR

The ^{1}H NMR of NRTOCl was performed in $CDCl_3$ at room temperature (Figure S-2). The expanded ^{1}H NMR of this compound shows that the most deshielded proton (H1) at 10.34 ppm is attributed to the hydrogen located on the pyridinium ring between positive nitrogen and amide group (Figure S-3). Because of the interaction between nitrogen lone pair and carbonyl of amide group, the chemical shifts of NH_2 protons are not equivalent in NRTOCl. In this compound one of the NH_2 protons appears at 9.86 ppm and another one is at 6.28 ppm. A doublet (J = 10 Hz) at 9.44 ppm is attributed to H5 located on the pyridinium ring in a position *ortho* to the positive nitrogen. The chemical shift of H3 in the *para* position with respect to the positive nitrogen appears 9.34 ppm as a doublet peak (J = 10 Hz). The final hydrogen on the pyridinium ring is H4 which appears as a triplet peak (J = 10 Hz) at 8.20 ppm. In the structure of NRTOCl there are four hydrogens on the ribose ring. The anomeric hydrogen (H1′) is impacted more by the oxygen atom of the ribose ring and the positive nitrogen of the pyridinium ring so that this hydrogen appears at 6.75 ppm as a doublet peak (J = 5 Hz). H2′ and H3′ are neighbors and appear as two triplet peaks (J = 5 Hz) with chemical shifts of 5.57 and 5.43 ppm respectively. Since H2′ is closer to the anomeric center than H3′, its chemical shift is more deshielded than that of H3′. A multiplet at 5.35 ppm with an integral of 6, confirms the presence of three H-C=C-H groups in the structure of NRTOCl. H4′ in the ribose ring and one of the hydrogens of the methylene group (H5′) bonded to the single oxygen of ester group overlap with each other and appear as a multiplet at 4.70 ppm with integral 2. Since the hydrogens of this methylene group are diastereotopic, another hydrogen of this methylene group appears at 4.50 ppm as a doublet of doublets (J_1= 14 Hz, J_2 = 4 Hz). In the three long chain ester groups of NRTOCl, there are three CH_2 groups near the ester carbonyl groups which appear as multiplets between 2.37-2.55 ppm and the six methylene groups in the proximity to the H-C=C-H groups appear at 2.02 ppm as a multiplet (Figure S-3). The multiplet at 1.63 ppm can be attributed to the other three methylene groups near the CH_2 groups bonded to the carbonyl groups. The broad peak at 1.30 ppm with an integral of 60 is attributed to the rest of 30 methylene groups. Finally, a triplet peak (J= 7.0 Hz) at 0.89 ppm with integral of 9 confirms the existence of three methyl groups at the end of the oleate esters arms.

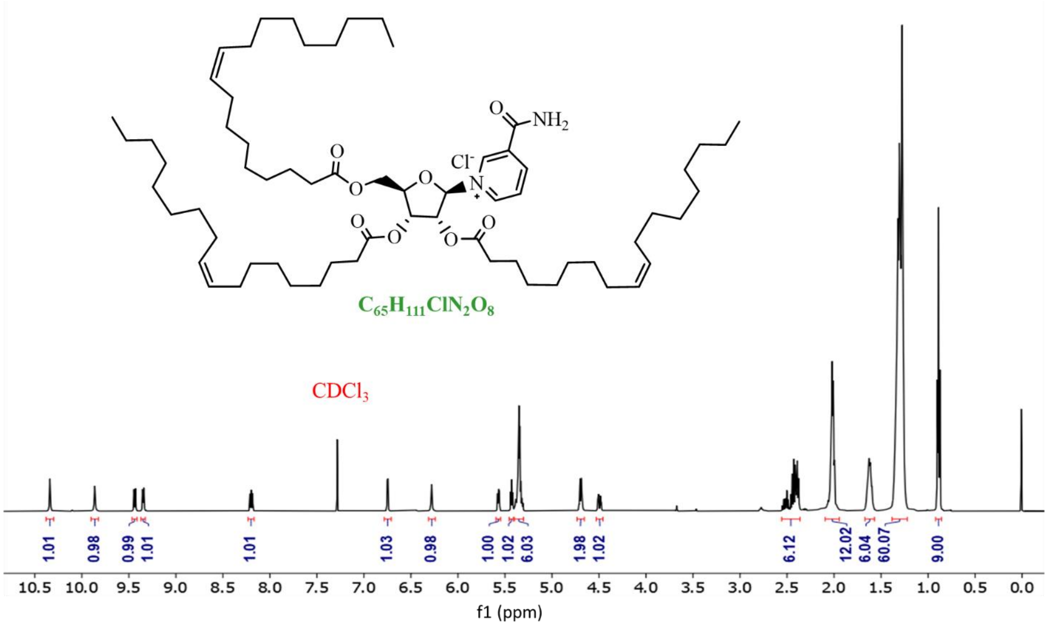

Figure S-2. ^{1}H NMR (500 MHz) of NRTOCl in $CDCl_3$.

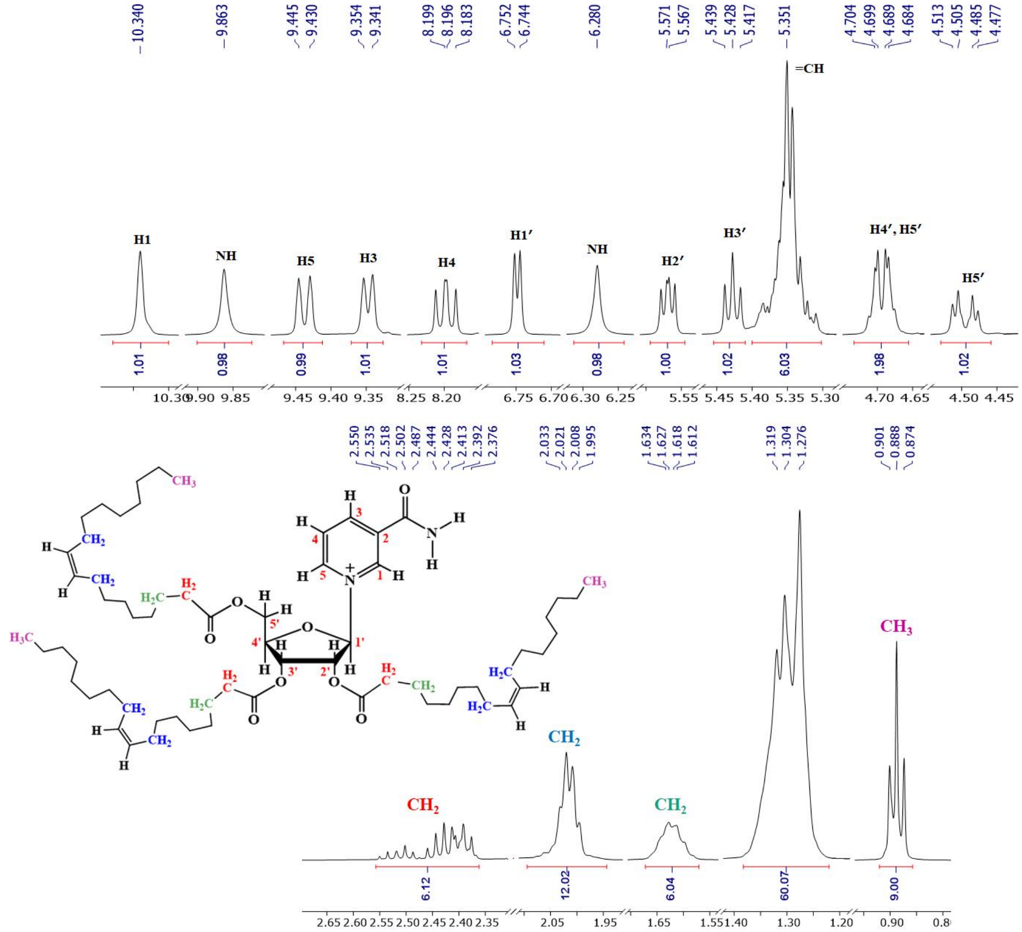

Figure S-3. Expanded ^{1}H NMR (500 MHz) of NRTOCl.

125 MHz ^{13}C NMR

The ^{13}C NMR (125 MHz) of NRTOCl in $CDCl_3$ was also studied at room temperature (Figure S-4). The expanded ^{13}C NMR of this compound exhibits three peaks at 173.1, 172.9 and 172.3 ppm attributed to the three different carbonyl carbons of the ester groups in the structure of NRTOCl (Figure S-5). A peak at 162.5 ppm confirms the carbonyl of the amide group in this compound. There are five distinct peaks at 146.7, 142.5, 141.8, 134.6 and 127.9 ppm for the carbons in the pyridinium ring. The chemical shifts of six carbons of three -C=C- groups are so close together and appear at 130.07, 130.06, 130.04, 129.67 and 129.62 ppm. Due to the close chemical shifts of these carbons, two carbons overlap and consequently, only five peaks for the alkene groups were observed. Four peaks at 98.0, 82.9, 75.8 and 69.1 ppm confirmed the existence of ribose ring in the structure of NRTOCl and the chemical shift of the methylene carbon bonded to the single oxygen of the ester group appears at 62.2 ppm. In the three long ester chains of NRTOCl, three distinct peaks at 33.9, 33.8 and 33.7 ppm are attributed to the three CH_2 groups near the carbonyl carbons of the ester groups (Figure S-5). The rest of the methylene groups in these chains appear between 22.7-31.9 ppm and because the chemical shifts of these carbons are close to each other, most of them overlap. We attributed the peak at 14.1 ppm to the three methyl groups at the end of the oleate chains.

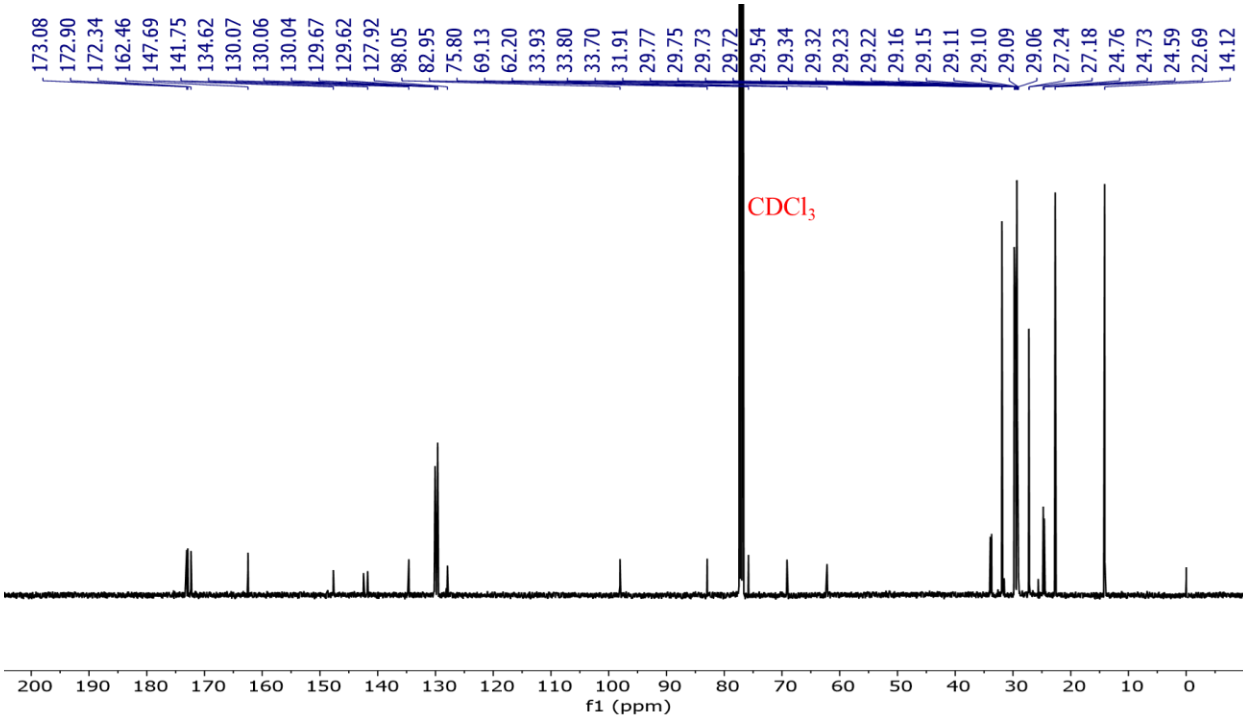

Figure S-4. ^{13}C NMR (125 MHz) of NRTOCl in $CDCl_3$.

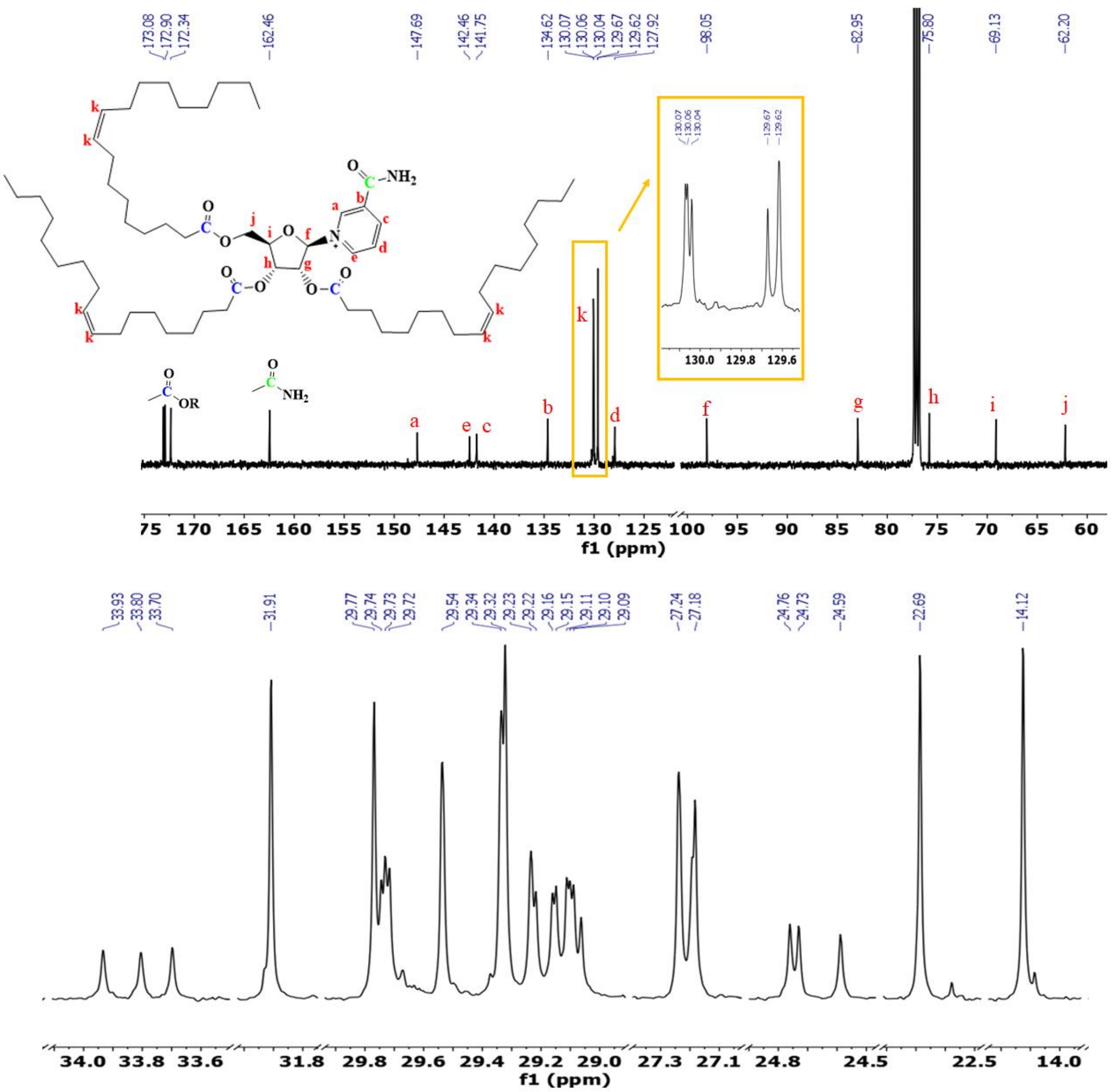

Figure S-5. Expanded ^{13}C NMR (125 MHz) in $CDCl_3$ of NRTOCl.

Liquid Chromatography-Mass Spectrometry

To confirm that there were three oleate groups, LC-MS was performed to find the molecular weight of NRTOCl (Figure S-6). The results of selected reaction monitoring (SRM) show a single peak with 1047.52 m/z (M-Cl) that is in agreement with the structure of NRTO cation. Interestingly, a fragment with 925.70 m/z is attributed to the ribose-trioleate molecule formed by elimination of nicotinamide molecule from NRTOCl.

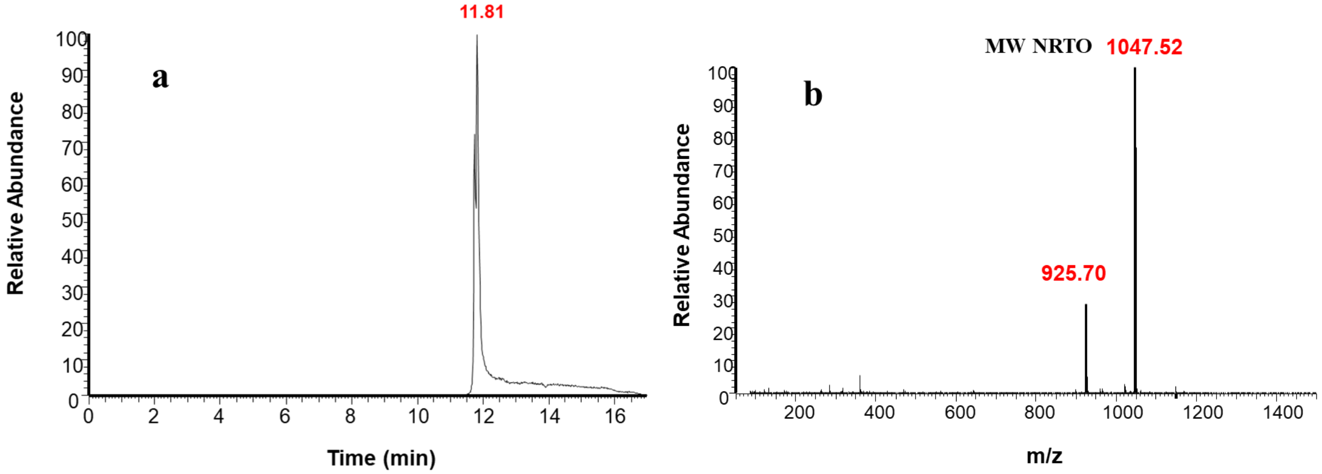

Figure S-6. SRM LC-MS of NRTOCl. a) SRM LC of NRTO cation. b) Mass spectrum of NRTO cation.

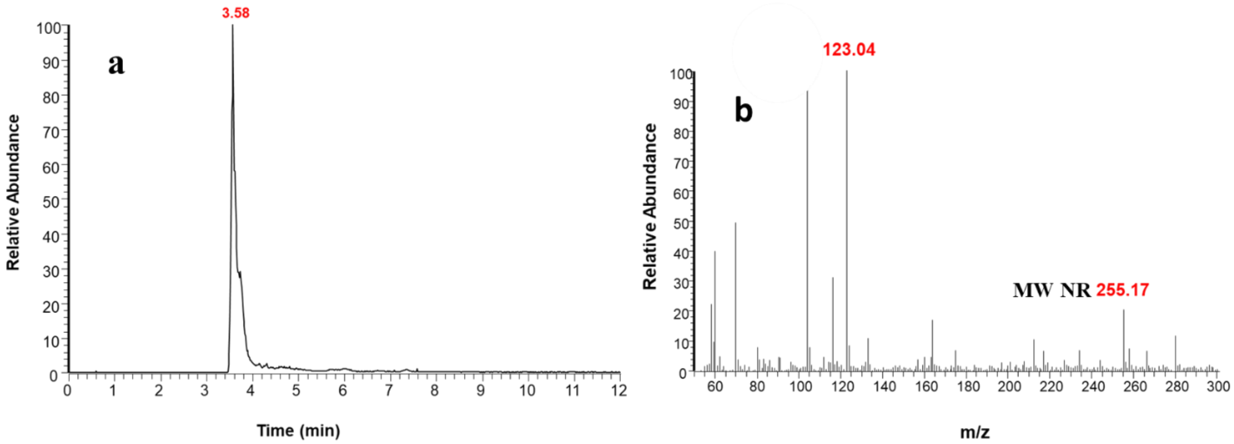

Figure S-7. a) SRM LC of released NR from NRTOCl, digested in the simulated intestinal phase. b) Mass spectrum of released NR from NRTOCl, digested in the simulated intestinal phase.
